# Asymmetrical Methylene-Bridge Linked Fully Iodinated Azoles as Energetic Biocidal Materials with Improved Thermal Stability

**DOI:** 10.3390/ijms241310711

**Published:** 2023-06-27

**Authors:** Xinyuan Zhao, Xun Zhang, Yan Liu, Siping Pang, Chunlin He

**Affiliations:** 1 School of Materials Science & Engineering, Beijing Institute of Technology, Beijing 100081, Chinazhangxun@bit.edu.cn (X.Z.); pangsp@bit.edu.cn (S.P.); 2 Experimental Center of Advanced Materials, School of Materials Science & Engineering, Beijing Institute of Technology, Beijing 100081, China; 3 Chongqing Innovation Center, Beijing Institute of Technology, Chongqing 401120, China; 4 Research Institute of Chemical Defense, Beijing 102205, China

**Keywords:** polyiodo compound, energetic materials, methylene bridging, biocidal materials

## Abstract

The instability and volatility of iodine is high, however, effective iodine biocidal species can be readily stored in iodinated azoles and then be released upon decomposition or detonation. Iodine azoles with high iodine content and high thermal stability are highly desired. In this work, the strategy of methylene bridging with asymmetric structures of 3,4,5-triiodo-1-H-pyrazole (TIP), 2,4,5-triiodo-1H-imidazol (TIM), and tetraiodo-1H-pyrrole (TIPL) are proposed. Two highly stable fully iodinated methylene-bridged azole compounds 3,4,5-triiodo-1-((2,4,5-triiodo-1H-imidazol-1-yl)methyl)-1H-pyrazole (**3**) and 3,4,5-triiodo-1-((tetraiodo-1H-pyrrol-1-yl)methyl)-1H-pyrazole (**4**) were obtained with high iodine content and excellent thermal stability (iodine content: 84.27% for compound **3** and 86.48% for compound **4**; T*_d_*: **3**: 285 °C, **4**: 260 °C). Furthermore, their composites with high-energy oxidant ammonium perchlorate (AP) were designed. The combustion behavior and thermal decomposition properties of the formulations were tested and evaluated. This work may open a new avenue to develop advanced energetic biocidal materials with well-balanced energetic and biocidal properties and versatile functionality.

## 1. Introduction

Biological organisms have always posed a threat to human beings, including but not limited to the novel coronavirus [1], variola virus (VARV) [2], or HIV [3]. As humans strive to combat viruses and bacteria, these pathogens continually mutate [4], making it incredibly challenging to eliminate them entirely [5,6,7,8,9,10]. Implementing necessary protective measures, such as elimination and isolation, can be effective [11,12,13]. Iodine has been proven to be a highly effective fungicide and can be used to disinfect air environments [14,15,16,17,18,19]. It can achieve a killing rate of 99.999% at 25 °C for a duration of 10 min under standard conditions, with intestinal bacteria, amoebic cysts, and enteroviruses requiring 0.2 ppm, 3.5 ppm, and 14.6 ppm iodine residues, respectively [20]. However, the instability and volatility of iodine make it inconvenient to be used directly as a biocidal agent under ambient conditions in its normal solid state.

The storage of iodine through forming C-I bonds in azole compounds and then releasing iodine upon decomposition or detonation is an effective way to stabilize iodine [21,22,23,24]. The fully iodinated single azole ring compounds, such as 3,4,5-triiodo-1-H-pyrazole (TIP, iodine content = 85.41%) [25], 2,4,5-triiodo-1H-imidazol (TIM, iodine content = 85.41%) [26], tetraiodo-1H-pyrrole (TIPL, iodine content = 88.95%), can readily achieve high iodine content (more than 80%) [21]. However, due to the existence of acidic N-H, they may suffer compatibility issues when formulating with other oxidizers to obtain improved oxygen balance in the consideration of practical applications, and their thermal stabilities still need to be improved; for instance, TIPL has poor thermal stability and has been observed to start decomposing at 168 °C.

**Figure 1:**
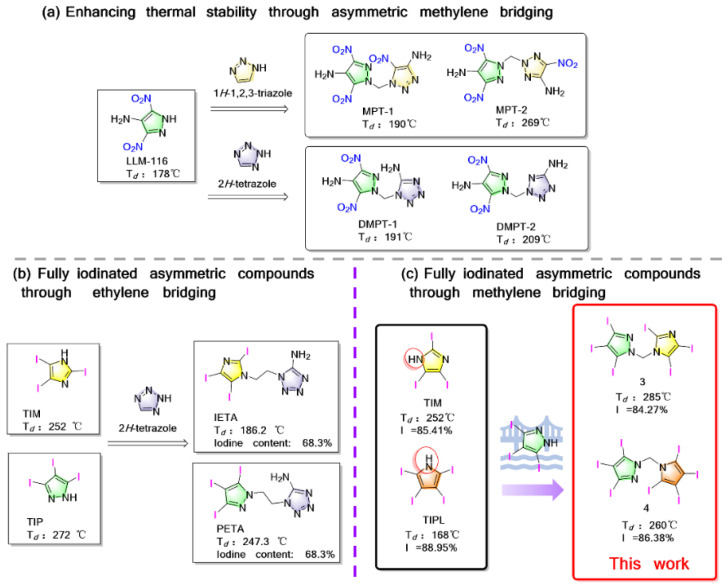
(**a**) Enhancing thermal stability through asymmetric methylene bridging energetic compounds. (**b**) Energetic derivation of asymmetric iodinated compounds based on ethylene bridging. (**c**) Fully iodinated asymmetric compounds through methylene bridging (this work).

The construction of a bridge connection (alkyl, C-N, C-C, N-N) is a well-established strategy to improve the thermal stability of single-ring compounds as well as eliminate acidic N-H, which are widely used in the field of energetic compounds [23,27,28,29,30]. A symmetric or asymmetric structure is always formed alternatively when a connecting bridge is introduced. Compared with a symmetric structure that is limited by the constraints of a single-ring molecular skeleton, an asymmetric structure can build more molecular skeletons by freely combining different skeletons, resulting in more diversified molecular structures to achieve optimal comprehensive performance [31,32,33,34,35,36,37,38,39,40,41,42]. The asymmetric bridge compounds based on oxadiazole rings through theoretical calculations and analyses demonstrate that the formation enthalpy, density, and detonation performance of the asymmetric compound are significantly improved [37]. Moreover, the effect of asymmetric structures on improving thermal stability have also been verified. The combination of pyrazole ring and 1,2,4-triazole ring, forming 3-(3,4-dinitro-1H-pyrazol-5-yl)-5-nitro-4H-1,2,4-triazole, has been observed to achieve a higher thermal decomposition temperature than the symmetrical bicyclic molecules [39]. Although 4-amino-3,5-dinitropyrazole (LLM-116) is a representative compound with a high density and low-impact sensitivity, its low decomposition temperature hinders its practical application. By methylene bridging with different ring skeletons (1,2,3-triazole or tetrazole), along with adjustable regional isomerization, it achieves higher thermal stability (T*_d_*: LLM-116: 178 °C, MPT-1: 190 °C, MPT-2: 269 °C, DMPT-1: 191 °C, DMPT-2: 209 °C) (Figure 1a) [40,41]. Based on N, N′ ethylene-bridging, energetic polyiodoazole compounds IETA and PETA were synthesized (Figure 1b) [42]; their iodine content decreased to a certain extent (iodine content: IETA = 68.3%, PETA = 68.3%), and due to the introduction of high-energy tetrazole, the thermal decomposition temperature of the compound decreased compared to the fully iodinated single-ring compound (T*_d_*: IETA: 186.2 °C, PETA: 247.3 °C).

In our continuing interests in developing novel polyiodoazole compounds, two methylene-bridged asymmetrical fully iodinated compounds, 3,4,5-triiodo-1-((2,4,5-triiodo-1H-imidazol-1-yl)methyl)-1H-pyrazole (**3**) and 3,4,5-triiodo-1-((tetraiodo-1H-pyrrol-1-yl)methyl)-1H-pyrazole (**4**), were designed and synthesized (Figure 1c). Their structures were characterized by NMR (nuclear magnetic resonance), IR (infrared radiation), EA (element analysis), HRMS (high resolution mass spectroscopy), and X-ray single crystal diffraction. Their properties were determined by DSC (differential scanning calorimetry) and thermochemical computer code. In order to evaluate their application potentials, the formulations of the prepared compounds with ammonium perchlorate (AP) were designed based on the zero oxygen balance formula. The composite samples of **3** and **4** with AP were prepared by mixing the components using mechanical grinding, and the composites were characterized by EDS (energy dispersive spectroscopy), and IR. Their combustion performances were tested by TGA (thermos gravimetric analyzer) and the hot spot test.

## 2. Results and Discussion

### 2.1. Synthesis

**Scheme 1:**
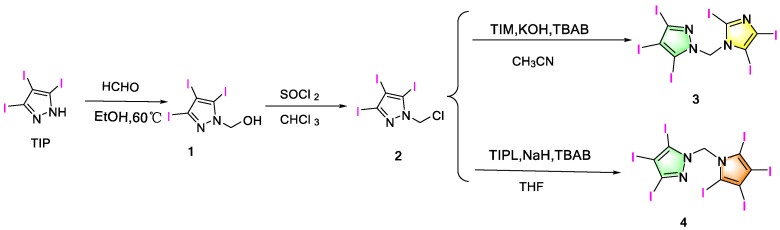
Synthesis route of compounds **3** and **4**.

The starting material (TIP, TIM, and TIPL) was prepared based on methods found in the literature [21,25,26]. The methylene bridge was introduced by reacting formaldehyde with TIP, resulting in the intermediate **1**, then it was treated with thionyl chloride in chloroform to give **2**, according to references (Scheme 1) [43]. Finally, **2** was reacted with TIM potassium salt or TIPL sodium salt in acetonitrile or tetrahydrofuran by adding a catalytic amount of tetrabutylammonium bromide (TBAB) as a phase-transfer catalyst to obtain **3** or **4** with a yield of 86% and 85% yield, respectively.

### 2.2. Single-Crystal X-ray Analysis

**Figure 2:**
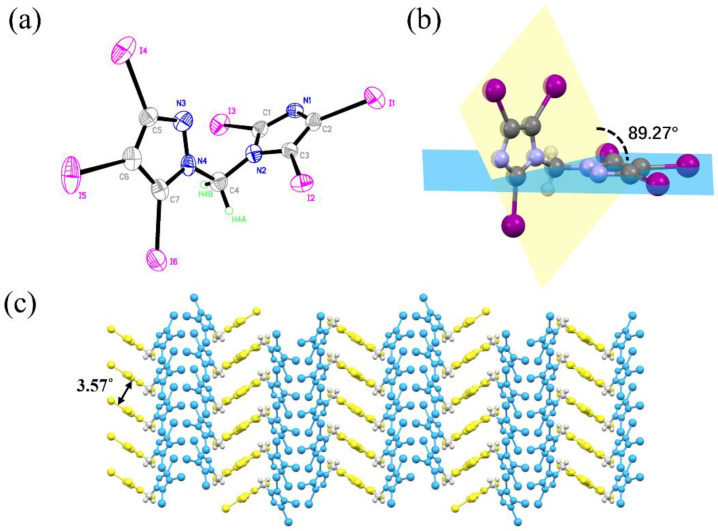
(**a**) The X-ray crystal structures of compound **3**. (**b**) The dihedral angle of compound **3**. (**c**) Capped sticks packing diagram of **3**.

The suitable single crystal of **3** was obtained by slow evaporation of **3** in a saturated DMSO solution. It crystallizes in monoclinic space group *Fdd2* with a crystal density of 3.606 g cm^−3^ at 150 K (Figure 2a and Table S1). The pyrazole ring and imidazole ring forms a dihedral angle of 89.27° (Figure 2b). A face-to-face stacking with a packing coefficient of 66.5% was observed in the packing diagram of **3**. The distance between the two imidazole layers is 3.57 Å, implying the existence of π-π interactions (Figure 2c).

### 2.3. Physicochemical and Energetic Properties

**Figure 3:**
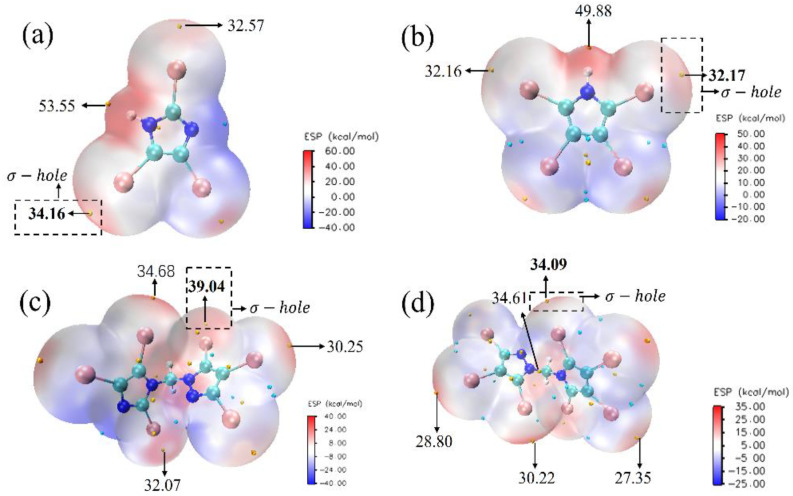
(**a**) Electrostatic potential surfaces (ESP) for TIM, (**b**) TIPL, (**c**) **3**, and (**d**) **4**. The blue and red spheres in the diagram represent the surface local minima and maximum of ESP, respectively.

The thermal stability of the prepared compounds was measured by DSC, as shown in Table 1, Figures S10 and S12. After linking by methylene bridge, the initial decomposition temperature (T_d_) of **3** and **4** was significantly improved, at 33 °C and 94 °C higher than TIM and TIPL, respectively (**3**: 285 °C, **4**: 260 °C Figure S13). In order to have a better understanding of the relationship between structure and properties, the analysis of surface electrostatic potential (ESP) was performed by Multiwfn v3.8 software to investigate the intermolecular halogen bond interactions [44]. The strength of the halogen bond is generally measured by the $V_{\left(s,max\right)}$ in the σ-hole region of the electrostatic potential (ESP) [45,46,47]. As shown in Figure 3a,b, the $V_{\left(s,max\right)}$ of single-ring TIM and TIPL is around the N-H atom, which is not favorable to stabilizing the molecule. In **3** and **4**, the $V_{\left(s,max\right)}$ is surrounding the pyrazole ring and the methylene bridge, and the $V_{\left(s,max\right)}$ values are slightly higher than that in TIM and TIPL, indicating that the strength of the halogen bond in a double-ring system is higher than that in a single-ring system (**3**: 39.04 kcal mol^−1^; **4**: 34.09 kcal mol^−1^, TIM: 34.16 kcal mol^−1^; TIPL: 32.17 kcal mol^−1^). These results are also consistent with the experimental results (T_d_: 3: 285 °C, 4: 260 °C, TIM: 252 °C, TIPL: 168 °C).

With high iodine content, the density of the compounds remains at a high level, with a density of 3.606 g cm^−3^ and 3.024 g cm^−3^ for **3** and **4**, respectively (Table 1 and Figure S13). The heats of formation (∆H*_f_*) were computed by Gaussian 09 software (see Supplementary Materials Scheme S1 and Table S2). Based on the heat formation enthalpy (∆H*_f_*) and density, the detonation velocities (*D*) and pressures (*P*) were calculated using the EXPLO5 (V6.05) program. It was observed that **3** and **4** have higher detonation pressures (6.77 GPa and 5.77 GPa, respectively) and detonation velocities (2574 and 2376 m s^−1^, respectively) than the fully iodinated single-ring compound (*D*: TIM: 4.37 GPa, TIPL: 3.27 GPa; *P*: TIM: 416.0 m s^−1^, TIPL: 449.9 m s^−1^), which contributes to achieving a wider range of sterilization.

**Figure 4:**
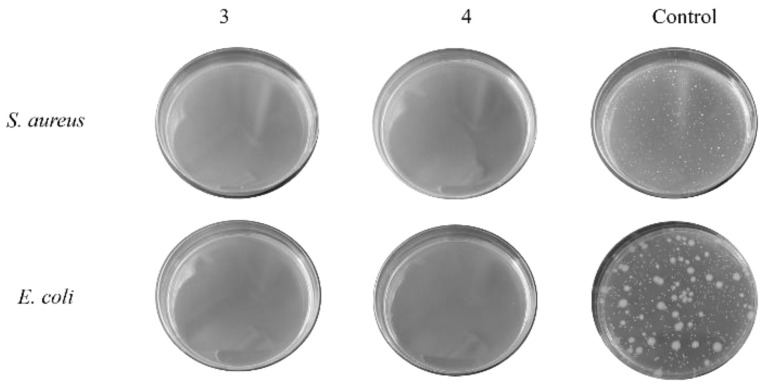
The growth of two strains of bacteria on the blank and sample-treated agar medium plates.

Microbiological tests were carried out to assess the bactericidal performances of compound **3** and **4** against two representative bacteria: Gram-positive *Staphylococcus aureus* (*S. aureus*) and Gram-negative *Escherichia coli* (*E. coli*). The sample was heated and burned by an alcohol lamp to generate smoke containing iodine in a small test tube (33 mL), then after waiting 5 s, the sample slowly and safely released large amounts of iodine vapor. The stained paper was placed around the mouth of the test tube, thereby receiving direct contact with the iodine vapor. Next, the treated stained paper was cultured overnight in Luria-Bertani (LB) medium at 37 °C in an incubator shaker, and then quantitative bactericidal performance was characterized by the plate count method. For comparison, the stained paper samples were immersed with liquid bacterial growth media and were directly cultured following the same procedure. The final concentration of bacteria was 5 × 10^5^~5 × 10^6^ CFU mL^−1^. The results showed that after a few seconds (5 s) in the presence of small doses of 5 mg, both compound **3** and **4** showed high antibacterial effects on the two bacteria, with an inhibition rate of ≥99.9% against *S. aureus* and *E. coli* (Figure 4).

### 2.4. Design and Tests of High Iodine Content Composites

**Figure 5:**
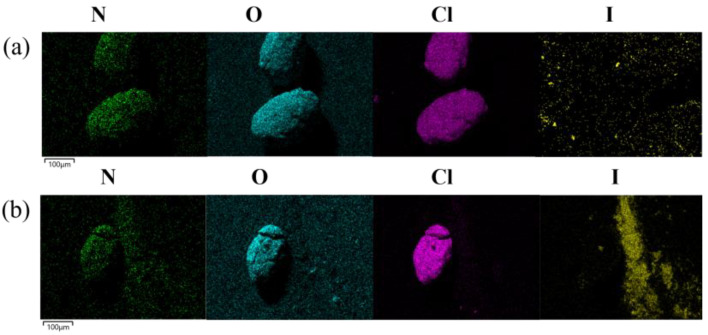
(**a**) The EDS images of F1. (**b**) The EDS images of F2.

In order to achieve complete combustion and a better sterilization effect, the mixing of fully iodinated compounds with ammonium perchlorate (AP) was designed and calculated. The oxygen balanced composite materials of **3**+AP (F1) and **4**+AP (F2) were designed to achieve zero oxygen balance (see Supplementary Materials Table S3). Based on the calculated results, formulations with high iodine contents of 47.33% (F1) and 48.61% (F2) of the prepared compounds with AP were produced by mechanical grinding. Energy dispersive spectroscopy (EDS) tests and IR (Figure S14) were performed to examine the element composition in both formulations. It can be seen from Figure 5a,b that all elements were detected on both formulations, indicating that finely mixed composites were achieved. A more uniform distribution of elemental iodine in **F1** demonstrated a better mixing of compound **3** and AP.

**Figure 6:**
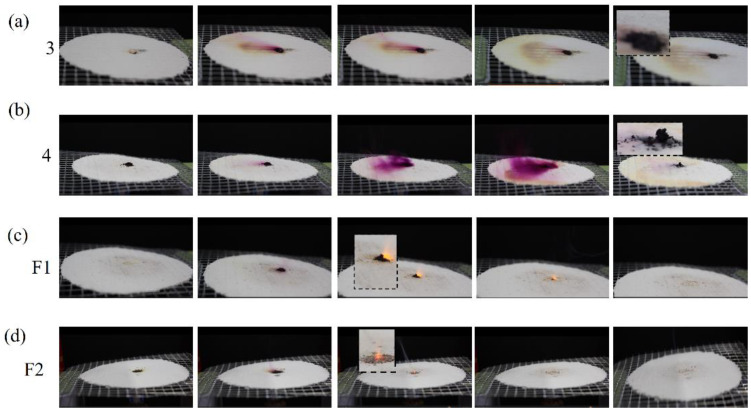
Hot spot test of compound **3** (**a**), **4** (**b**), formula F1 (**c**), and F2 (**d**).

The thermal decomposition processes of F1 and F2 were studied by differential scanning calorimetry (DSC) and thermogravimetric analysis (TGA). They showed a thermal decomposition temperature above 260 °C, indicating good thermal stability (F1: 289.34 °C, F2: 265.11 °C) (see Supplementary Materials Figures S15 and S16). In the TG curve (see Supplementary Materials Figure S17), both composites showed that a one-stage weight loss started at 220 °C, with a weight loss of 97.45% for composite F1 and 94.60% for F2. Compared to the neat samples of **3** or **4**, a larger weight loss was observed after mixing with AP, especially for **3**, where more than 17% weight loss was achieved (see Supplementary Materials). The decomposition process of **3** and **4** and their composition with AP were tested by placing the samples on an asbestos mesh under heating. As shown in Figure 6, the decomposition process of **3** and **4** was mild and generated continuous smoking, with a large amount of remaining black residue observed. On the other hand, for the decomposition process of the composite materials containing AP, a complete decomposition was achieved and small amounts of solid residues were observed. Additionally, a flame was observed during the composition process of both of the composite materials containing AP, demonstrating that the formula composite material has better redox reactions and generates more gas products that are beneficial for distributing the iodine species to a larger area.

## 3. Materials and Methods

### 3.1. General Methods

^1^H and ^13^C NMR spectra were tested using a Bruker 400 MHz spectrometer (400 and 100 MHz, respectively) in *d*-DMSO. Chemical shifts are reported as δ values relative to internal standard *d*-DMSO (δ 2.50 for ^1^H NMR and 39.52 for ^13^C NMR). Infrared spectra (IR) were obtained on a PerkinElmer Spectrum BX FT-IR instrument equipped with an ATR unit at 25 °C. Elemental analyses of C/H/N/I were investigated on a Vario EL III Analyzer. The onset decomposition temperature was measured using a TA Instruments DSC25 differential scanning calorimeter at a heating rate of 5 °C min^−1^ under dry nitrogen atmosphere. Densities were determined at room temperature by a Micromeritics AccuPyc 1340 gas pycnometer.

Fully iodinated monocyclic compound precursors 3,4,5-triiodo-1H-pyrazole (TIP), 2,4,5-triiodo-1H-imidazole (TIM), and 2,3,4,5-tetraiodo-1H-pyrrole (TIPL) were synthesized as references [16,20,21].

### 3.2. (3,4,5-Triiodo-1H-pyrazol-1-yl) Methanol (***1***)

To a 250 mL round bottle flask, 3,4,5-triiodo-1H-pyrazole (TIP) (4.46 g, 10 mmol) was dissolved in 52 mL of ethanol. Subsequently, formaldehyde (HCHO, 37%) (10 mmol) was added. The resulting mixture was heated at 60 °C for 1 h, then the reaction temperature was slowly decreased to room temperature and reacted for another 48 h. The resulting solution was poured into ice water (100 g). A white precipitate was deposited. The solid was collected by filtration, and dried to yield 3.2 g of white solid compound **1** (67%). ^1^H NMR (*d*_6_-DMSO): δ 7.04 (s, 1H), 5.46 (s, 2H) ppm. ^13^C NMR (*d*_6_-DMSO): δ 108.97, 97.80, 88.07, 75.64 ppm. IR (KBr pellet) υ 3069.74, 2943.57, 2807.72, 1427.48, 1384.11, 1313.03, 1290.81, 1270.44, 1186.61, 1071.79, 1040.75, 979.49, 961.46, 747.47, 648.17, 627.89, 509.87, 450.26, 418.64 cm^−1^. EA (C_4_H_3_N_2_I_3_O, 475.74): Calcd (%), C, 10.10; H, 0.64; I, 80.02; N, 5.89; O, 3.36; Found (%), C, 10.00; H, 0.54; I, 80.23; N, 5.68; O, 3.26 (Figures S1 and S2).

To a 250 mL round bottle flask, 3,4,5-triiodo-1H-pyrazole (TIP) (4.46 g, 10 mmol) was dissolved in 52 mL of ethanol. Subsequently, formaldehyde (HCHO, 37%) (10 mmol) was added. The resulting mixture was heated at 60 °C for 1 h, then the reaction temperature was slowly decreased to room temperature and reacted for another 48 h. The resulting solution was poured into ice water (100 g). A white precipitate was deposited. The solid was collected by filtration, and dried to yield 3.2 g of white solid compound **1** (67%). ^1^H NMR (*d*_6_-DMSO): δ 7.04 (s, 1H), 5.46 (s, 2H) ppm. ^13^C NMR (*d*_6_-DMSO): δ 108.97, 97.80, 88.07, 75.64 ppm. IR (KBr pellet) υ 3069.74, 2943.57, 2807.72, 1427.48, 1384.11, 1313.03, 1290.81, 1270.44, 1186.61, 1071.79, 1040.75, 979.49, 961.46, 747.47, 648.17, 627.89, 509.87, 450.26, 418.64 cm^−1^. EA (C_4_H_3_N_2_I_3_O, 475.74): Calcd (%), C, 10.10; H, 0.64; I, 80.02; N, 5.89; O, 3.36; Found (%), C, 10.00; H, 0.54; I, 80.23; N, 5.68; O, 3.26 (Figures S1 and S2).

### 3.3. 1-(Chloromethyl)-3,4,5-triiodo-1H-pyrazole (***2***)

The compound **1** (3.5 g, 7.36 mmol) was dissolved in 18 mL of ethanol, then 4 mL of SOCl_2_ was added dropwise. The reaction mixture was stirred at 60 °C for 10 h, then solvent was removed under vacuum to yield 2.8 g of white solid compound **2** (78%). ^1^H NMR (*d*_6_-DMSO): δ 6.18(s, 2H) ppm. ^13^C NMR (*d*_6_-DMSO): δ 112.37, 100.78, 90.67, 60.17 ppm. IR (KBr pellet) υ 3039.54, 2979.94, 1443.21, 1425.28, 1392.40, 1301.87, 1280.19, 1146.90, 1029.23, 976.55, 921.38, 755.35, 672.84, 647.88, 606.94, 482.61, 426.82 cm^−1^. EA (C_4_H_2_ClN_2_I_3_, 493.70): Calcd (%), C, 9.72; H, 0.41; Cl, 7.17; I, 77.03; N, 5.67; Found (%), C, 9.62; H, 0.45; Cl, 7.23; I, 77.12; N, 5.57 (Figures S3 and S4).

### 3.4. 3,4,5-Triiodo-1-((2,4,5-triiodo-1H-imidazol-1-yl)methyl)-1H-pyrazole (***3***)

TIM (0.18 g, 0.4 mmol) was dissolved in 5 mL of anhydrous acetonitrile, then KOH (0.025 g 0.44 mmol) was added. The mixture was stirred at room temperature until a clear solution was achieved. The excess KOH was filtered off, then **2** (0.25 g, 0.5 mmol) was added to the above reaction mixture, followed by adding tetrabutylammonium bromide (TBAB) (0.103 g, 0.32 mmol). The mixture was heated to 75 °C and refluxed for another 12 h. After the reaction mixture was cooled to room temperature, a white solid was deposited and collected by filtration to yield 0.31 g of white solid compound **3** (86%). ^1^H NMR (*d*_6_-DMSO): δ 6.23 (s, 2H) ppm. ^13^C NMR (*d*_6_-DMSO): δ 110.64, 100.63, 99.87, 98.23, 89.35, 88.71, 66.13 ppm. IR (KBr pellet) υ 1456.49, 1443.19, 1377.62, 1354.67, 1303.11, 1280.68, 1228.02, 1199.28, 1170.36, 997.15, 968.47, 948.04, 795.73, 757.07, 424.83 cm^−1^. EA (C_7_H_2_N_4_I_6_, 903.45 amu): Calcd (%), C, 9.31; H, 0.22; I, 84.27; N, 6.20; Found (%), C, 9.61; H, 0.32; I, 84.37; N, 6.44. HRMS (ESIMS): calcd for C_7_H_3_N_4_I_6_^+^ [M+H] ^+^ 904.4620, found 904.4621 (Figures S5, S6 and S9).

### 3.5. 3,4,5-Triiodo-1-((periodo-1H-pyrrol-1-yl) methyl)-1H-pyrazole (***4***)

TIPL (0.21 g, 0.37 mmol) was dissolved in 5 mL of anhydrous tetrahydrofuran, then NaH (9.3 mg, 0.44 mmol) was added, and the mixture was stirred at 60 °C for 30 min. After the reaction mixture was cooled down to room temperature, **2** (0.228 g, 0.46 mmol) and tetrabutylammonium bromide (TBAB) (0.095 g, 0.30 mmol) were added to the mixture. After heating at 75 °C for another 12 h, the reaction mixture was slowly cooled to room temperature, a white solid was deposited and was collected by filtration to yield 0.32 g of white solid compound 4 with a yield of 85%. ^1^H NMR (*d*_6_-DMSO): δ 6.33 (s, 2H) ppm. ^13^C NMR (*d*_6_-DMSO): δ 110.21, 99.44, 91.12, 88.60, 87.78, 70.81 ppm. IR (KBr pellet) υ 1445.20, 1332.52, 1299.27, 1264.36, 1230.06, 1116.57, 965.67, 759.75, 623.02, 602.98, 494.37cm^−1^. EA (C_8_H_2_N_3_I_7_, 1028.36): Calcd (%), C, 9.34; H, 0.20; I, 86.38; N, 4.09; Found (%), C, 9.44; H, 0.30; I, 86.58; N, 4.19. HRMS (ESIMS): calcd for C_8_H_3_N_3_I_7_^+^ [M+H] ^+^ 1029.3634, found 1029.3638 (Figures S7, S8 and S11).

## 4. Conclusions

In conclusion, two new asymmetric fully iodinated compounds (**3** and **4**) were designed and synthesized. Their iodine contents were maintained at a high level with the elimination of the acidic N-H and their thermal stability was improved compared to their precursors TIM and TIPL (**3**: iodine content = 84.27%, T*_d_*: 285 °C) (**4**: iodine content = 86.38%, T*_d_*: 260 °C). Their structures and properties were fully characterized (via NMR, IR, HRMS, and EA), and the structure of **3** was further determined by single-crystal X-ray diffraction. The electrostatic potential (ESP) calculations show the existence of strong intermolecular bonds (e.g., halogen bonds), which help to achieve better thermal stability. Additionally, the compositions of **3** or **4** with AP were designed, and their properties were evaluated. Two formulations possessing zero oxygen balance with high iodine content (F1: iodine content % = 47.33%; F2: iodine content % = 48.61%) were prepared and their decomposition behaviors were tested and analyzed. More complete combustion and fewer solid residues were observed, thus demonstrating their great promise as biocidal composites.

## Figures and Tables

**Table 1:** Physicochemical and energetic properties of compounds **1**, **2**, **3**, and **4**.

Compd	*T_m_^a^* (°C)	*T_d_^b^* (°C)	*d ^c^* (g cm^−3^)	Δ*H_f_^d^* (kJ mol^−1^)	*D ^e^* (m s^−1^)	*P ^f^* (Gpa)	Iodine Content (%)
**3**	244	285	3.606	676.4	2574	6.77	84.27
**4**	187	260	3.024	967.8	2376	5.77	86.38
TIM	191	252	3.27	416.0	2646	4.37	85.41
TIPL	-	168	3.62	449.9	2253	3.27	88.95

*^a^* Melting point temperature from DSC (5 °C min^−1^). *^b^* Decomposition temperature from DSC (5 °C min^−1^). *^c^* Density measured using a gas pycnometer at 25 °C. *^d^* Heat of formation—Gaussian 09 (Revision E.01). *^e^* Detonation velocity—EXPLO5 V6.05. *^f^* Detonation pressure—EXPLO5 V6.05.

## Data Availability

Not applicable.

## Supplementary Materials

The following supporting information can be downloaded at: https://www.mdpi.com/article/10.3390/ijms241310711/s1: X-ray crystallographic data, copies of 1H-NMR and 13C-NMR spectra, IR and DSC Figure of compound **3** and **4**, DFT Calculations and Design, calculation and characterization of composite materials, references [48,49,50,51,52,53,54,55] are cited in there.

## References

[1] Budd J., Miller B.S., Manning E.M., Lampos V., Zhuang M., Edelstein M., Rees G., Emery V.C., Stevens M.M., Keegan N. (2020). Digital technologies in the public-health response to COVID-19. Nat. Med..

[2] Mühlemann B., Vinner L., Margaryan A., Wilhelmson H., Castro C.d.l.F., Allentoft M.E., Damgaard P.d.B., Hansen A.J., Nielsen S.H., Strand L.M. (2020). Diverse variola virus (smallpox) strains were widespread in northern Europe in the Viking Age. Science.

[3] Bershteyn A., Kim H.Y., Braithwaite R.S. (2022). Real-Time Infectious Disease Modeling to Inform Emergency Public Health Decision Making. Annu. Rev. Public Health.

[4] Geller C., Varbanov M., Duval R.E. (2012). Human Coronaviruses: Insights into Environmental Resistance and Its Influence on the Development of New Antiseptic Strategies. Viruses.

[5] Wang C.C., Prather K.A., Sznitman J., Jimenez J.L., Lakdawala S.S., Tufekci Z., Marr L.C. (2021). Airborne transmission of respiratory viruses. Science.

[6] Yougbare S., Mutalik C., Okoro G., Lin I.H., Krisnawati D.I., Jazidie A., Nuh M., Chang C.C., Kuo T.R. (2021). Emerging Trends in Nanomaterials for Antibacterial Applications. Int. J. Nanomed..

[7] Mutalik C., Saukani M., Khafid M., Krisnawati D.I., Widodo, Darmayanti R., Puspitasari B., Cheng T.-M., Kuo T.-R. (2023). Gold-Based Nanostructures for Antibacterial Application. Int. J. Mol. Sci..

[8] Mutalik C., Okoro G., Krisnawati D.I., Jazidie A., Rahmawati E.Q., Rahayu D., Hsu W.-T., Kuo T.-R. (2021). Copper sulfide with morphology-dependent photodynamic and photothermal antibacterial activities. J. Colloid Interface Sci..

[9] Mutalik C., Wang D.-Y., Krisnawati D.I., Jazidie A., Yougbare S., Kuo T.-R. (2020). Light-Activated Heterostructured Nanomaterials for Antibacterial Applications. Nanomaterials.

[10] Zhong Y., Zheng X.T., Zhao S., Su X., Loh X.J. (2022). Stimuli-Activable Metal-Bearing Nanomaterials and Precise On-Demand Antibacterial Strategies. ACS Nano.

[11] Shen W. (2021). Dynamically adjusted strategy in response to developments in the COVID-19 pandemic as a new normal. Glob. Heal..

[12] Nardell E.A., Nathavitharana R.R. (2020). Airborne Spread of SARS-CoV-2 and a Potential Role for Air Disinfection. JAMA.

[13] Noorimotlagh Z., Jaafarzadeh N., Martinez S.S., Mirzaee S.A. (2021). A systematic review of possible airborne transmission of the COVID-19 virus (SARS-CoV-2) in the indoor air environment. Environ. Res..

[14] Dev Kumar G., Mishra A., Dunn L., Townsend A., Oguadinma I.C., Bright K.R., Gerba C.P. (2020). Biocides and Novel Antimicrobial Agents for the Mitigation of Coronaviruses. Front. Microbiol..

[15] Bloomfield S.F., Megid R. (1994). Interaction of iodine with Bacillus subtilis spores and spore forms. Appl. Bacteriol..

[16] Wei D., Xue Y.-Q. (2011). Research Progress of Iodine Fungicides. Liaoning Chem. Ind..

[17] Li Q., Korza G., Setlow P. (2017). Killing the spores of *Bacillus* species by molecular iodine. J. Appl. Microbiol..

[18] Chen P., Dou H., Fei T., He C., Pang S. (2018). Research Progress in Iodine-based Energetic Biocidal Agents. Chin. J. Energetic Mater..

[19] Sun Y., Wang X., Fan L., Xie X., Miao Z., Ma Y., He T., Zha Z. (2020). Facile synthesis of monodisperse chromogenic amylose–iodine nanoparticles as an efficient broad-spectrum antibacterial agent. J. Mater. Chem. B.

[20] Kaiho T. (2015). Iodine Chemistry and Applications.

[21] He C., Zhang J., Shreeve J.M. (2013). Dense iodine-rich compounds with low detonation pressures as biocidal agents. Chemistry.

[22] Chand D., He C., Hooper J.P., Mitchell L.A., Parrish D.A., Shreeve J.M. (2016). Mono- and diiodo-1,2,3-triazoles and their mono nitro derivatives. Dalton Trans..

[23] He C., Hooper J.P., Shreeve J.M. (2016). Iodine-Rich Imidazolium Iodate and Periodate Salts: En Route to Single-Based Biocidal Agents. Inorg. Chem..

[24] Zhao G., He C., Kumar D., Hooper J.P., Imler G.H., Parrish D.A., Shreeve J.M. (2019). 1,3,5-Triiodo-2,4,6-trinitrobenzene (TITNB) from benzene: Balancing performance and high thermal stability of functional energetic materials. Chem. Eng. J..

[25] Chand D., Shreeve J.M. (2015). Versatile polyiodopyrazoles: Synthesis and biocidal promise. Chem. Commun..

[26] Katritzky A.R., Cundy D.J., Chen J. (1993). Polyiodoimidazoles and their nitration products. Energetic-Mater..

[27] He C., Zhao G., Hooper J.P., Shreeve J.M. (2017). Energy and Biocides Storage Compounds: Synthesis and Characterization of Energetic Bridged Bis(triiodoazoles). Inorg. Chem..

[28] Chinnam A.K., Shlomovich A., Shamis O., Petrutik N., Kumar D., Wang K., Komarala E.P., Tov D.S., Sućeska M., Yan Q.L. (2018). Combustion of energetic iodine-rich coordination polymer—Engineering of new biocidal materials. Chem. Eng. J..

[29] Zhao G., He C., Zhou W., Hooper J.P., Imler G.H., Parrish D.A., Shreeve J.M. (2018). Control of Biohazards: A High Performance Energetic Polycyclized Iodine-Containing Biocide. Inorg. Chem..

[30] Chang J., He C., Pang S., Shreeve J.M. (2022). N, N’-methylene-bridged nitroiodoazoles: Biocidal compounds with enhanced thermal stability. Chem. Eng. J..

[31] Kumar D., Mitchell L.A., Parrish D.A., Shreeve J.N.M. (2016). Asymmetric N,N′-ethylene-bridged azole-based compounds: Two way control of the energetic properties of compounds. J. Mater. Chem. A.

[32] Tang Y., He C., Imler G.H., Parrish D.A., Shreeve J.N.M. (2016). Design and synthesis of N-methylene-C linked tetrazole and nitramino-1,2,4-triazole: An approach to promising energetic materials. J. Mater. Chem. A.

[33] Kumar D., Imler G.H., Parrish D.A., Shreeve J.M. (2017). N-Acetonitrile Functionalized Nitropyrazoles: Precursors to Insensitive Asymmetric N-Methylene-C Linked Azoles. Chemistry.

[34] Kumar D., Imler G.H., Parrish D.A., Shreeve J.N.M. (2017). Aminoacetonitrile as precursor for nitrogen rich stable and insensitive asymmetric N-methylene-C linked tetrazole-based energetic compounds. J. Mater. Chem. A.

[35] Liu Y., He C., Tang Y., Imler G.H., Parrish D.A., Shreeve J.M. (2018). Asymmetric nitrogen-rich energetic materials resulting from the combination of tetrazolyl, dinitromethyl and (1,2,4-oxadiazol-5-yl)nitroamino groups with furoxan. Dalton. Trans..

[36] Yan T., Yang C., Ma J., Cheng G., Yang H. (2022). Intramolecular integration of multiple heterocyclic skeletons for energetic materials with enhanced energy & safety. Chem. Eng. J..

[37] Jin X., Xiao M., Zhou J., Zhou G., Hu B. (2019). Design of Energetic Materials Based on Asymmetric Oxadiazole. Chemistryopen.

[38] Kumar D., Imler G.H., Parrish D.A., Shreeve J.N.M. (2017). Resolving synthetic challenges faced in the syntheses of asymmetric N, N′-ethylene-bridged energetic compounds. New J. Chem..

[39] Cai J., Xie C., Xiong J., Zhang J., Yin P., Pang S. (2022). High performance and heat-resistant pyrazole-1,2,4-triazole energetic materials: Tuning the thermal stability by asymmetric framework and azo-bistriazole bridge. Chem. Eng. J..

[40] Xiong J., Cai J., Lai Q., Yin P., Pang S. (2022). Asymmetric assembly of pyrazole and 1,2,3-triazole with a methylene bridge: Regioisomerism and energetic properties. Chem. Commun..

[41] Xiong J., Chang J., Cai J., Yin P., Pang S. (2022). N-Functionalization of 5-Aminotetrazoles: Balancing Energetic Performance and Molecular Stability by Introducing ADNP. Int. J. Mol. Sci..

[42] Zhao G., Kumar D., He C., Hooper J.P., Imler G.H., Parrish D.A., Shreeve J.M. (2017). New Generation Agent Defeat Weapons: Energetic N,N′-Ethylene-Bridged Polyiodoazoles. Chemistry.

[43] Elguero J. (1986). A Selective synthesis of unsymmetrical 1.1′-netxylenebisdiazoles by solid-liquid phase transfer catalysis. Heterocycles.

[44] Lu T., Chen F. (2012). Multiwfn: A multifunctional wavefunction analyzer. J. Comput. Chem. Comput. Chem..

[45] Cavallo G., Metrangolo P., Milani R., Pilati T., Priimagi A., Resnati G., Terraneo G. (2016). The Halogen Bond. Chem. Rev..

[46] Yeo C.I., Tan Y.S., Kwong H.C., Lee V.S., Tiekink E.R.T. (2022). I⋯N halogen bonding in 1 : 1 co-crystals formed between 1,4-diiodotetrafluorobenzene and the isomeric n-pyridinealdazines (n = 2, 3 and 4): Assessment of supramolecular association and influence upon solid-state photoluminescence properties. CrystEngComm.

[47] Bennion J.C., Vogt L., Tuckerman M.E., Matzger A.J. (2016). Isostructural Cocrystals of 1,3,5-Trinitrobenzene Assembled by Halogen Bonding. Cryst. Growth Des..

[48] Spackman M.A., McKinnon J.J. (2002). Fingerprinting intermolecular interactions in molecular crystals. CrystEngComm.

[49] Spackman M.A., Jayatilaka D. (2009). Hirshfeld surface analysis. CrystEngComm.

[50] Becke A.D. (1992). Density-functional thermochemistry. III. The role of exact exchange. J. Chem. Phys..

[51] Stephens P.J., Chabalowski C., Jakanen K.J. (1994). Ab initio calculation of vibrational circular dichroism spectra using large basis set MP2 force fields. Chem. Phys. Lett..

[52] Stromberg A., Gropen O., Wahlgren U. (1982). Gaussian basis sets for the fourth-row main group elements, In–Xe. Comput. Chem..

[53] Glukhovtsev M.N., Pross A. (1995). Extension of Gaussian2 (G2) theory to bromine and iodinecontaining molecules: Use of effective core potentials. J. Chem. Phys..

[54] Westwell M.S., Searle M.S., Wales D.J., Williams D.H. (1995). Empirical Correlations between Thermodynamic Properties and Intermolecular Forces. J. Am. Chem. Soc..

[55] Tang J., Bai W. (1992). Prescription Design and Optimization of the Multi-Component mixed explosives. Explos. Mater..

