# Supplementary material for: Asymmetrical Methylene-Bridge Linked Fully Iodinated Azoles as Energetic Biocidal Materials with Improved Thermal Stability
Source: Int J Mol Sci. 2023 Jun 27;24(13):10711. doi: 10.3390/ijms241310711 (PMC10341448; doi:10.3390/ijms241310711)
Supplement: Supplementary file 1 [file ijms-24-10711-s001.zip › ijms-2453076-supplementary.pdf]

Supporting Information (SI)

Asymmetrical methylene bridged linked fully iodinated azoles as energetic biocidal materials with improved thermal stability

Xinyuan Zhao [1], Xun Zhang [1,2,3], Yan Liu [4,*], Siping Pang [1] and Chunlin He [1,2,3,*]

1 School of Materials Science & Engineering, Beijing Institute of Technology, Beijing 100081, China

2 Experimental Center of Advanced Materials, School of Materials Science & Engineering, Beijing Institute of Technology, Beijing 100081, China.

3 Chongqing Innovation Center, Beijing Institute of Technology, Chongqing 401120, China.

4 Research Institute of Chemical Defense, Beijing 102205, China

* Correspondence: liuyanwbl@163.com (Y. L); pangsp@bit.edu.cn (S.P.); chunlinhe@bit.edu.cn (C.H.);

Research Institute of Chemical Defense, Beijing 102205, China

Contents

Ⅰ. X-Ray crystallographic data 1

II.Copies of ^{1}H-NMR and ^{13}C-NMR spectra 2

III. IR and DSC Figure of compound 3 and 4 6

IV.DFT Calculations 8

V. Design, calculation and characterization of composite materials 10

Ⅰ. X-Ray crystallographic data

Table S1. Crystal data and structure refinement for **3.**

	**3**
CCDC No.	2258820
Empirical Formula	$C_7H_2I_6N_4$
Formula Weight	903.53
Temperature (K)	150K
Crystal System	orthorhombic
Space group	*Pdd2*
b (Å)	8.5935(5)
c (Å)	14.0338(4)
α (°)	90
β (°)	90
γ (°)	90
Volume (Å^3)	6656.3(7)
Z	16
Density (g cm^{-3}) (calculated)	3.606
F(000)	6240.0
F(000)	6197.31
Crystal size (mm^3)	0.15 × 0.08 × 0.05
Goodness-of-fit on F^2	1.052
Final R indexes [I>=2σ (I)]	R1 = 0.0389, wR2 = 0.1031
Final R indexes [all data]	R1 = 0.0436, wR2 = 0.1071

Ⅱ.Copies of ^{1}H-NMR and ^{13}C-NMR spectra

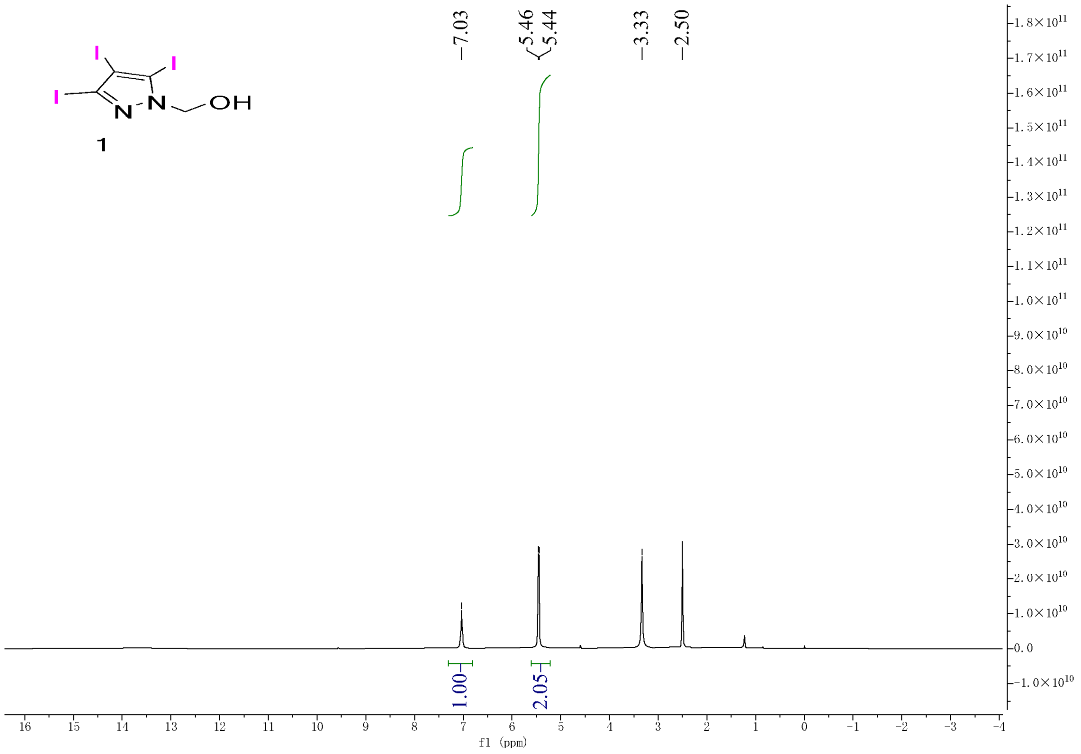

Figure S1. ^{1}H NMR spectrum of **1** in d_6-DMSO.

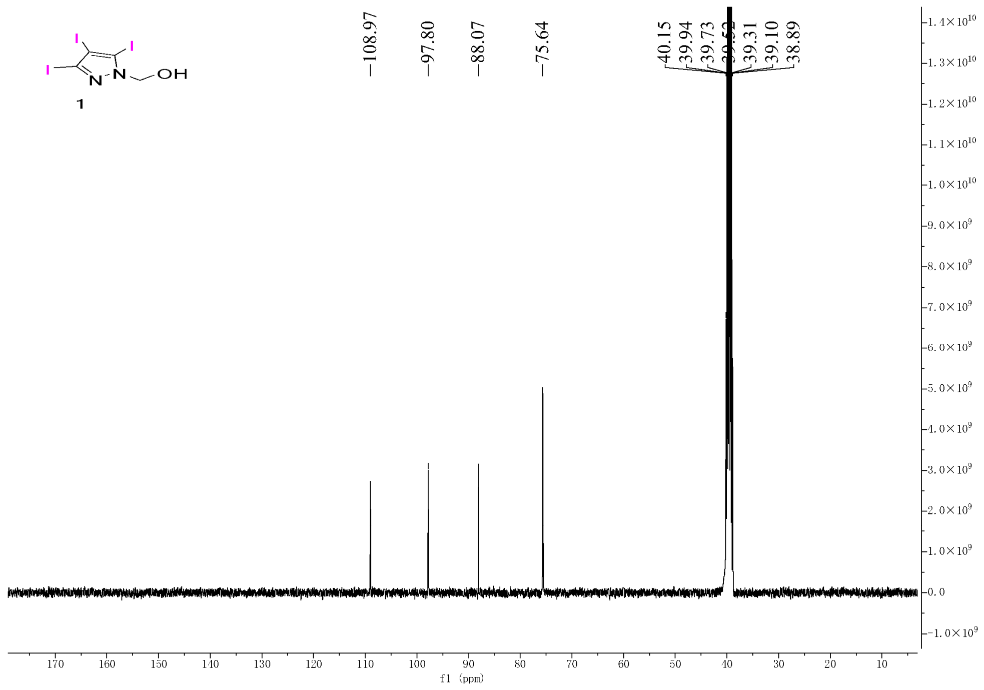

Figure S2. ^{13}C NMR spectrum of **1** in d_6-DMSO.

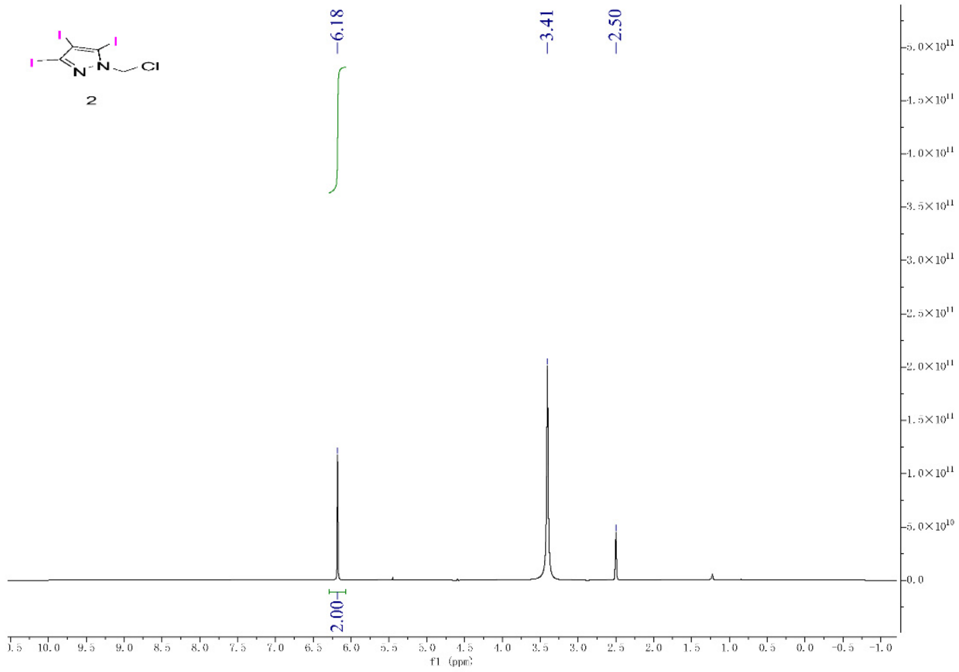

Figure S3. ^{1}H NMR spectrum of **2** in d_6-DMSO.

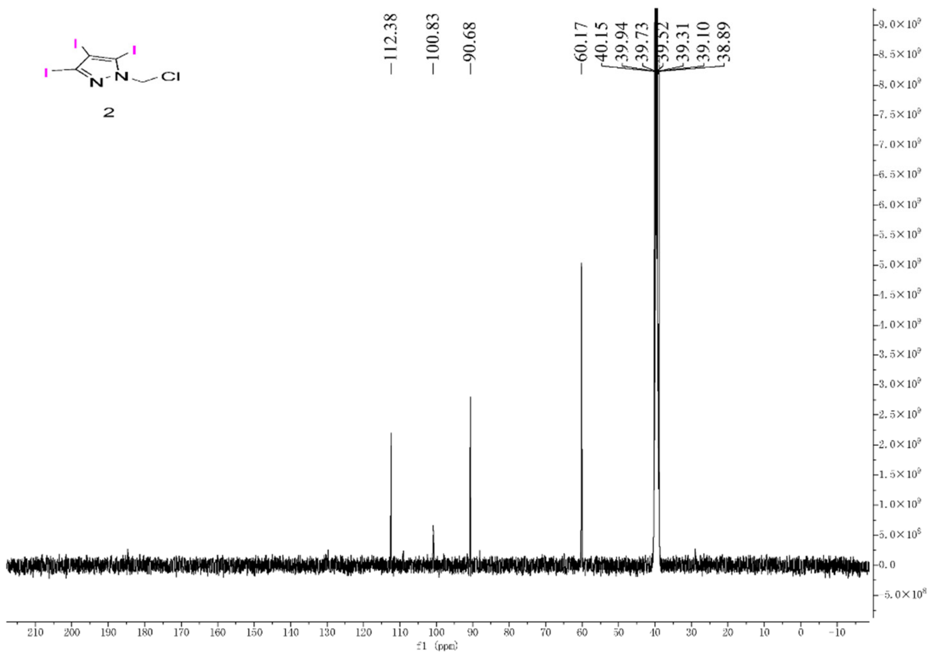

Figure S4. ^{13}C NMR spectrum of **2** in d_6-DMSO.

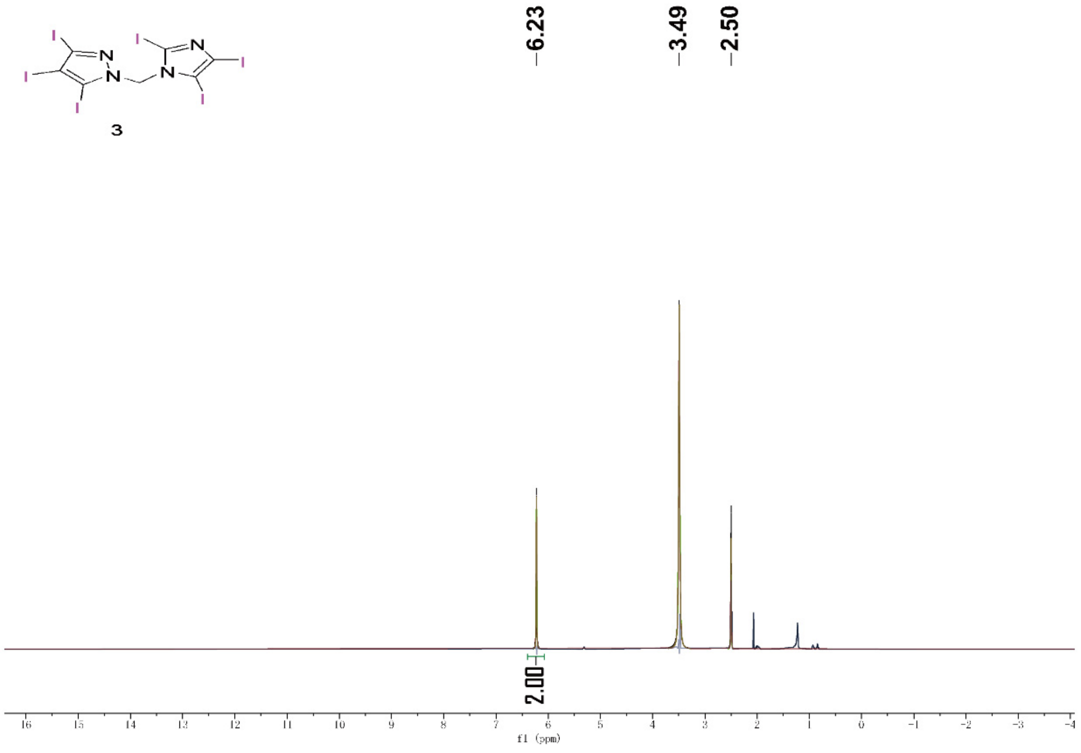

Figure S5. ^{1}H NMR spectrum of **3** in d_6-DMSO.

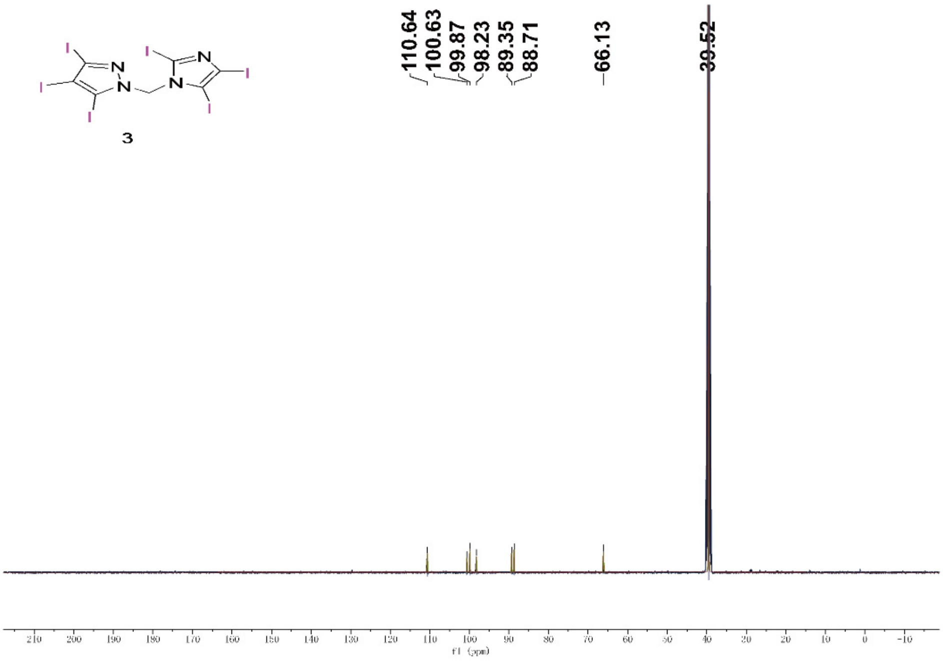

Figure S6. ^{13}C NMR spectrum of **3** in d_6-DMSO.

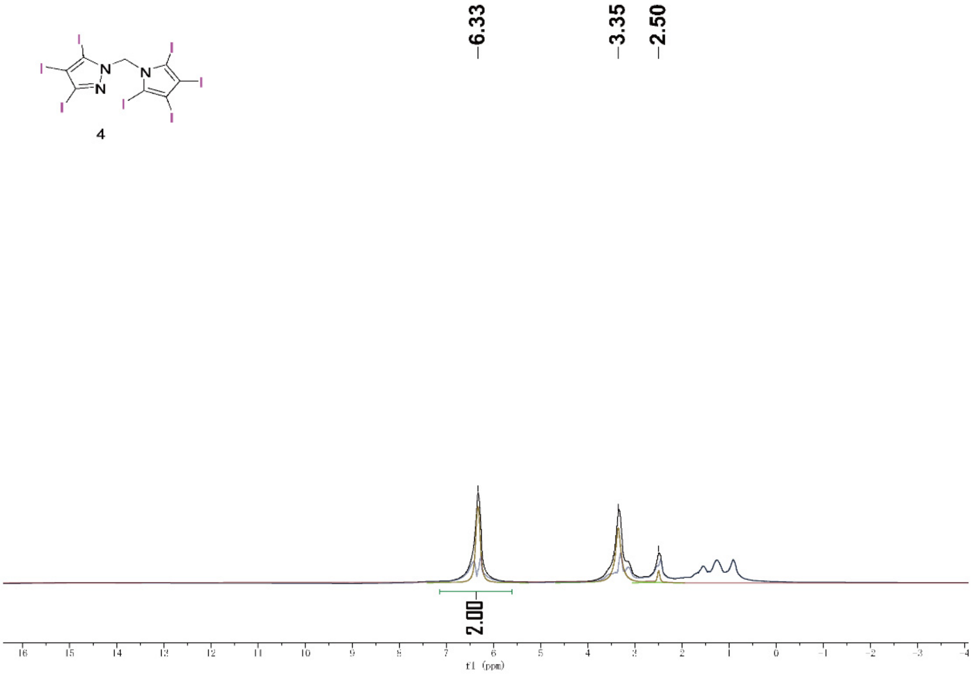

Figure S7. ^{1}H NMR spectrum of **4** in d_6-DMSO.

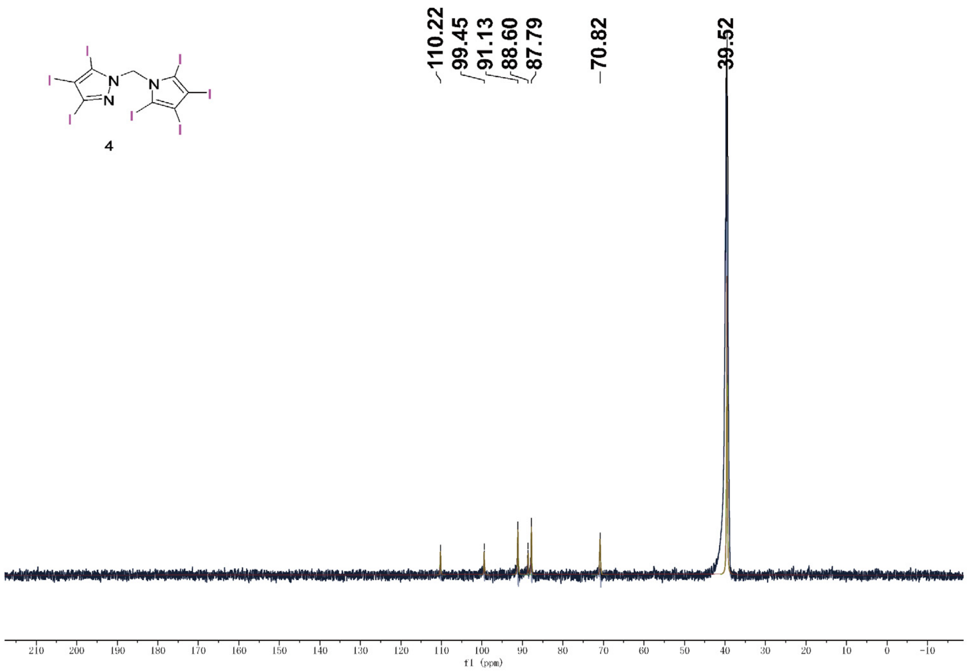

Figure S8. ^{13}C NMR spectrum of **4** in d_6-DMSO.

Ⅲ. IR and DSC Figure of compound 3 and 4

The initial thermal decomposition temperatures (T_d) of **3** and **4** were investigated by differential scanning calorimetry (DSC) at the temperature of 5℃min^{-1} in nitrogen atmosphere.

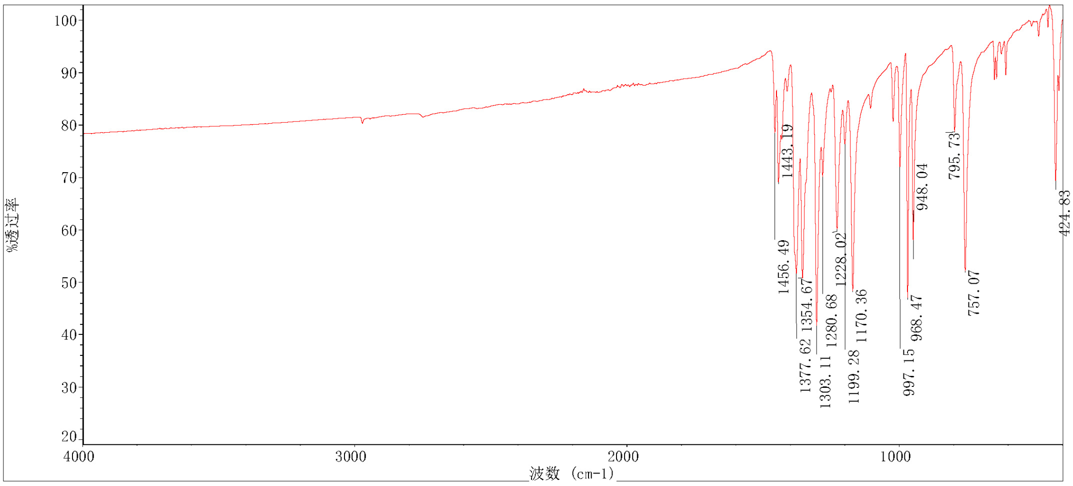

Figure S9. IR spectrum of compound **3**

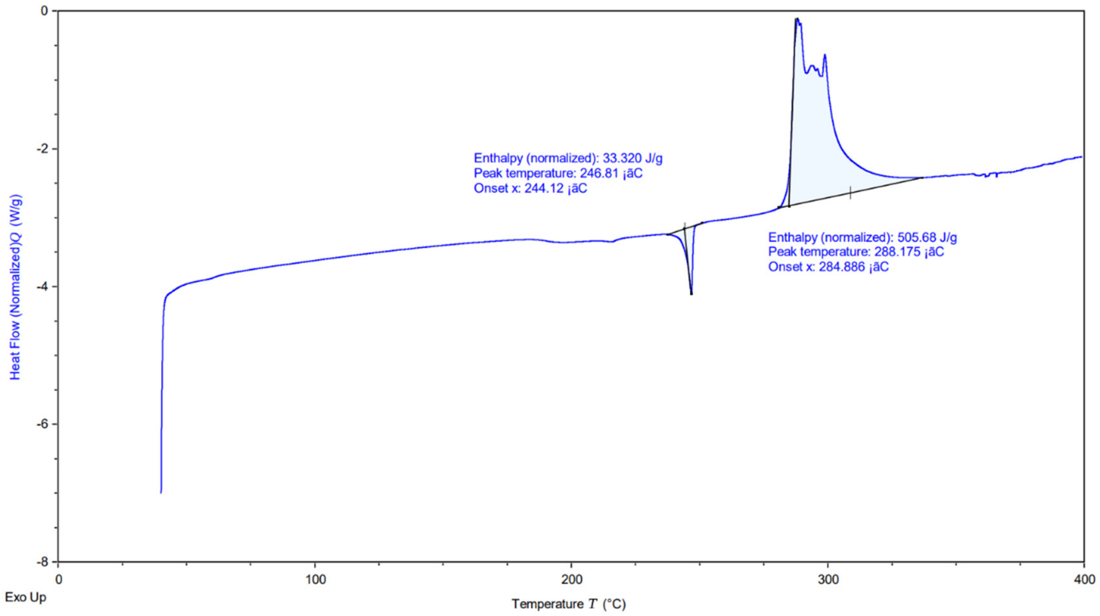

Figure S10. DSC scan of compound **3** at 5 °C min^{-1}

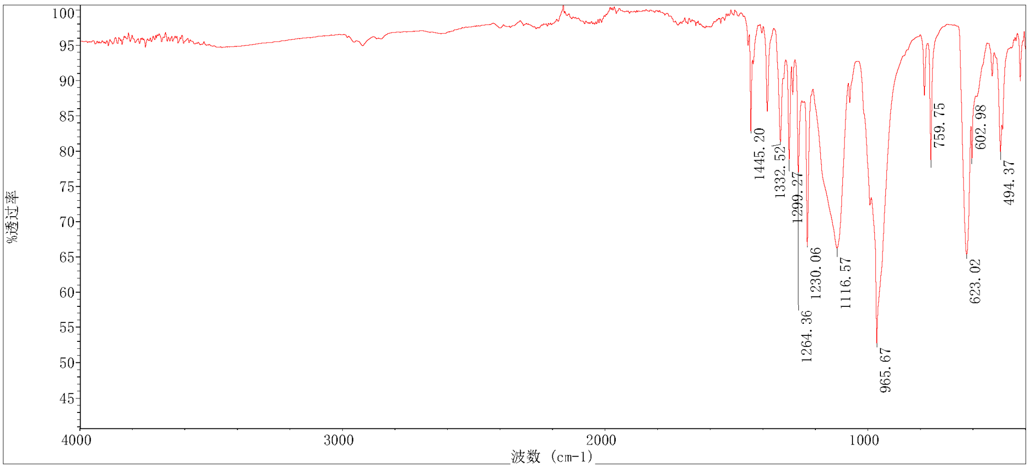

Figure S11. IR spectrum of compound **4**

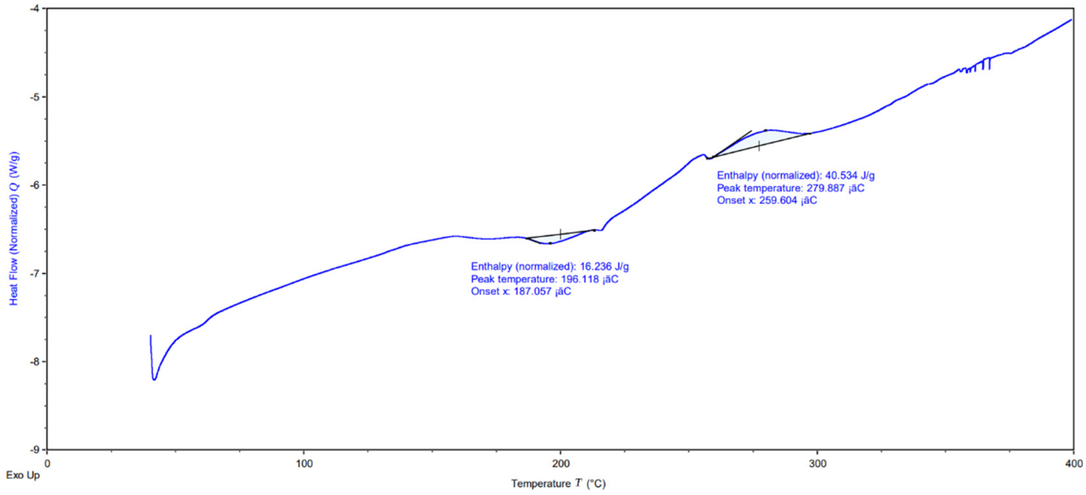

Figure S12. DSC scan of compound **4** at 5 °C min^{-1}

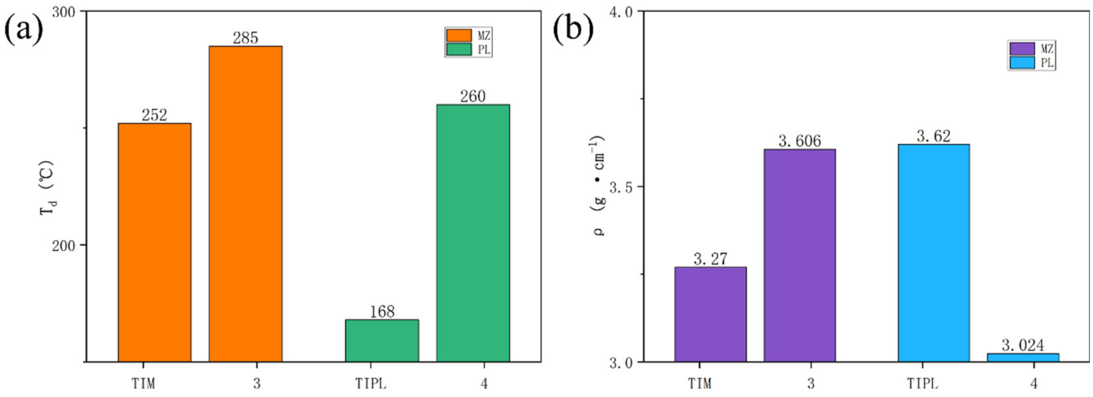

Figure S13. The T*d* of compounds TIM, TIPL, **3** and **4** (**a**), the density of TIM, TIPL, **3** and **4** (**b**).

Ⅳ.DFT Calculations

Theoretical calculations were performed by using the Gaussian 09 suite of programs[50, 51]. Single point energy (SPE) refinement on the optimized geometries was performed with the use of M062X/def2tzvp level. For the iodine-rich compounds, the def2 series basis sets that are based Stromberg et al., are used for optimization[52]. The enthalpy of reaction was carried out by combining the M062X/def2tzvp energy difference for the reactions[53], the scaled zero-point energies (ZPE), values of thermal

correction (HT) and other thermal factors. The gas phase heats of formation for four compounds were obtained by using isodesmic reactions. All of the structures were characterized to be true local energy minima on the potential energy surface without imaginary frequencies. The heats of formation in the solid state were further obtained by using Trouton's rule according to equation (1) (T represents either melting point or decomposition temperature when no melting occurs prior to decomposition) [54].

$$\Delta H_{sub} = 188/J\ mol^{-1}\ K^{-1} \times T \qquad \text{equation (1)}$$

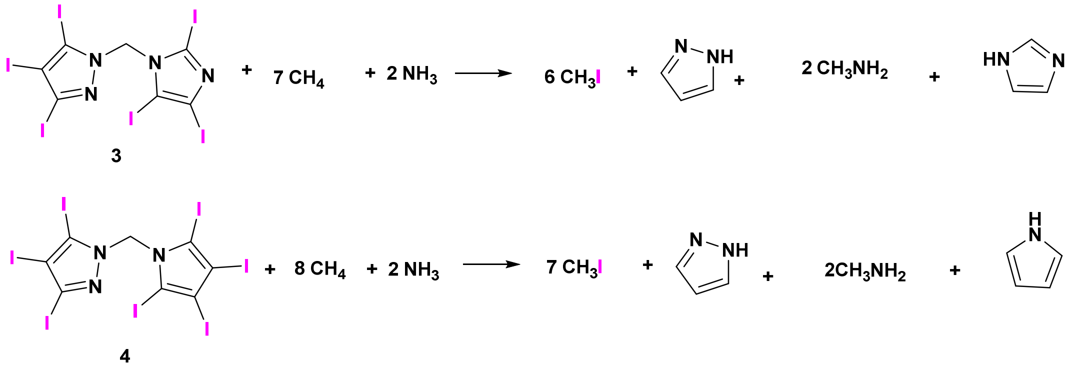

Scheme S1. Isodesmic reactions for **3** and **4**.

Table S2. Calculated total energy (E_0), zero-point energy (ZPE), values of the correction (H_{corr}), and enthalpy of formation in gas-state (HOF) for **3** and **4**.

**Compound**	**ZPE (a.u.)**	**H_{corr} (a.u.)**	**E_0 (a.u.)**	**Corrected E_0**	**$\Delta_f H_{gas}$ (kJ mol^{-1})**	ΔH_{sub}	**$\Delta_f H_{solid}$ (kJ mol^{-1})**
CH_4	0.044451	0.048269	-40.450124	-40.40363304	-74.6 [a]		
CH_3I	0.036296	0.040423	-337.4713491	-337.4323779	14.6 [a]		
NH_3	0.034034	0.037844	-56.4731351	-56.43665246	-45.9 [a]		
CH_3NH_2	0.063602	0.067952	-95.7246632	-95.65925528	-23 [a]		
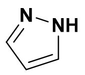	0.071405	0.076042	-225.9329976	-225.8598118	177.4 [a]		
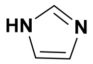	0.071293	0.075953	0.06844128	0.00466	136.1597878		
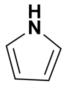	0.082558	0.087426	0.07925568	0.004868	143.2		
**3**	0.087908	0.107609	-2272.168955	-2272.06486	678.439	10.85	676.399
**4**	0.088596	0.110385	-2553.083904	-2552.97706	965.152	-14.15	967.812

[a] Data from NIST WebBook

Ⅴ. Design, calculation and characterization of composite materials

Oxygen balance calculation of composite materials is calculated refer to related literature[55]

$$OB = \sum_{i=1}^{n} OB_i W_i$$

Oxygen balance CaHbOcNdIe: $OB_{CO2} = 1600(c-b/2-2*a)/MW$

Table S3. Ratio and iodine content under zero oxygen balance of formulas

Formula	Ratio (polyiodide : AP)	Iodine content
3+AP (F1)	56 : 44	47.33%
4+AP (F2)	56 : 44	48.61%

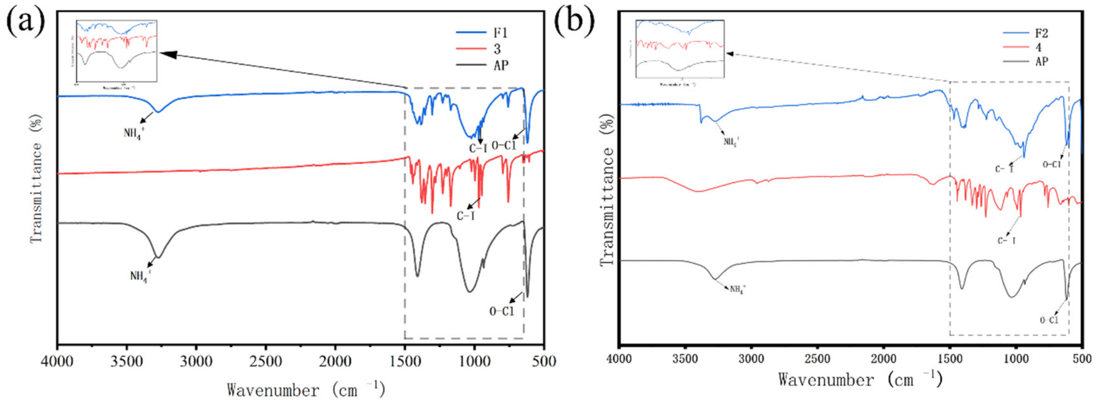

Figure S14. FTIR spectra of **3**, AP and F1 (**a**), and **4**, AP and F2 (**b**).

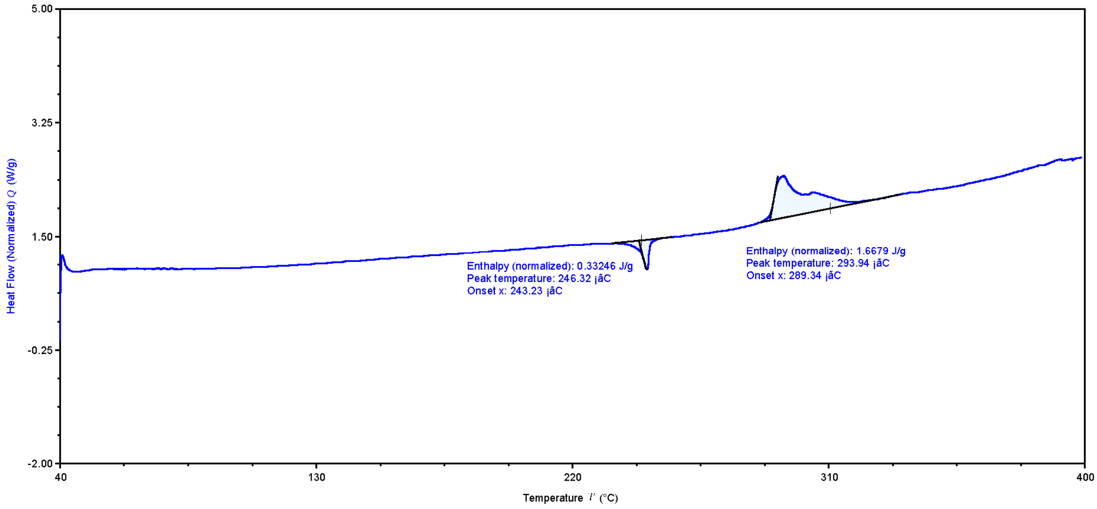

Figure S15. DSC scan of composite F1

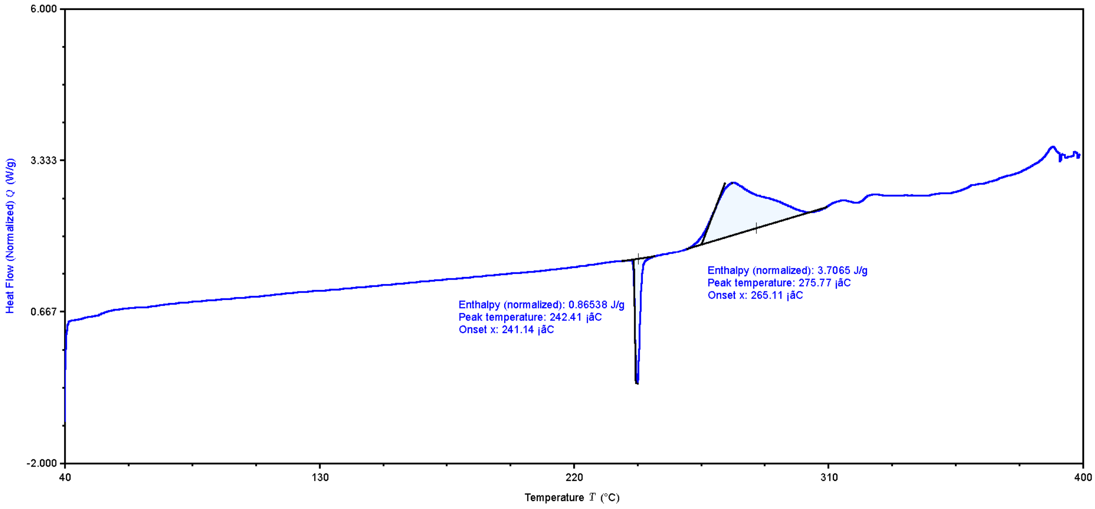

Figure S16. DSC scan of composite F2

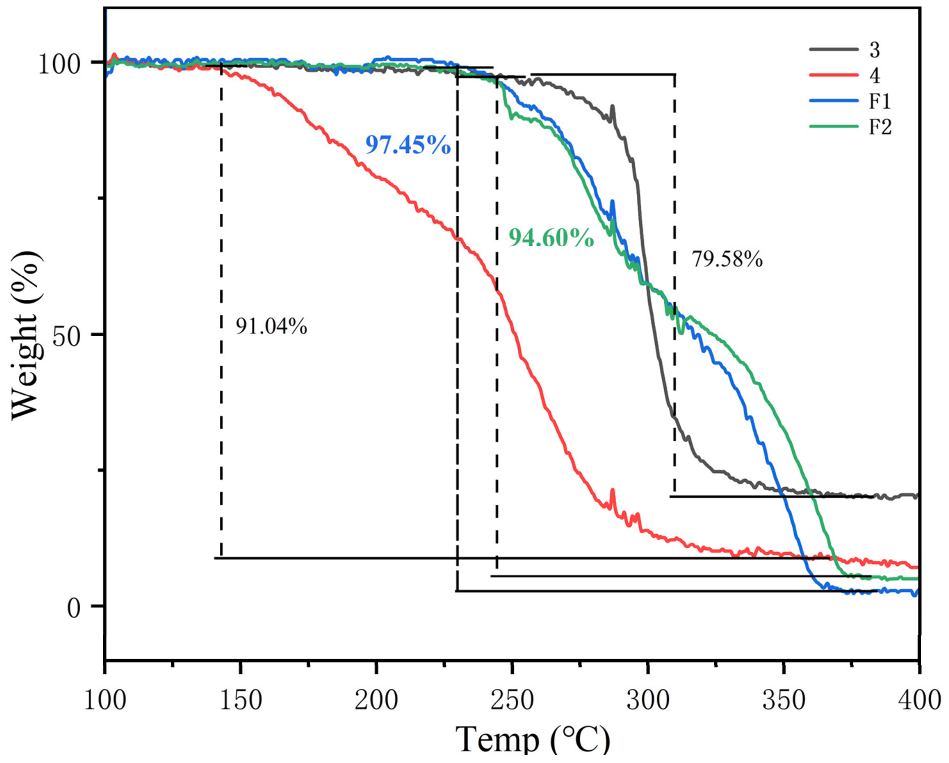

Figure S17. TGA traces for **3**, **4** compound and F1, F2 composites. Samples are heated in nitrogen at 5 ℃/min

References

48. A, M.; Spackman; McKinnon, J. J. Fingerprinting intermolecular interactions in molecular crystals. CrystEngComm, 2002, 66.

49. Spackman, M. A.; Jayatilaka, D. Hirshfeld surface analysis. CrystEngComm, 2009, 11.

50. Becke, A. D. Density-functional thermochemistry. III. The role of exact exchange. J. Chem. Phys, 1992.

51. Stephens, P. J.; Chabalowski, C.; a, F. J. D.; Jakanen, K. J. Ab initio calculation of vibrational circular dichroism spectra using large basis set MP2 force fields Chemical Physics Letters, 1994.

52. Stromberg, A.; Gropen, O.; Wahlgren, U. Gaussian basis sets for the fourth-row main group elements, In–Xe. Computational Chemistry, 1982, 4.

53. N, M.; Glukhovtsev; Pross, A. Extension of Gaussian2 (G2) theory to bromine and iodinecontaining molecules: Use of effective core potentials. J. Chem. Phys, 1995.
54. S, M.; Westwell; Searle, M. S.; Wales, D. J.; Williams, D. H. Empirical Correlations between Thermodynamic Properties and Intermolecular Forces. Journal of the american chemical society, 1995, 117.
55. Tang, J.; Bai, W. Prescription Design and Optimization of the Multi-Component mixed explosives. Explosive Materials, 1992, 70.
